# Predicting the Invasion Risk of the Highly Invasive *Acacia mearnsii* in Asia under Global Climate Change

**DOI:** 10.3390/plants13202846

**Published:** 2024-10-11

**Authors:** Anil Poudel, Pradeep Adhikari, Prabhat Adhikari, Sue Hyuen Choi, Ji Yeon Yun, Yong Ho Lee, Sun Hee Hong

**Affiliations:** 1 Department of Plant Resources and Landscape Architecture, College of Agriculture and Life Sciences, Hankyong National University, Anseong 17579, Republic of Korea; aneeelily@gmail.com (A.P.); adprabhat2051@gmail.com (P.A.); yun1230300@naver.com (J.Y.Y.); 2 Institute of Humanities and Ecology Consensus Resilience Lab, Hankyong National University, Anseong 17579, Republic of Korea; pdp2042@gmail.com; 3 OJeong Resilience Institute, Korea University, Seoul 02841, Republic of Korea

**Keywords:** *Acacia mearnsii*, invasive weed, climate change, invasion risk, managing invasive species, MaxEnt analysis

## Abstract

*Acacia mearnsii*, among the 100 worst invasive weeds worldwide, negatively impacts native biodiversity, agriculture, and natural ecosystems. Global climate change, characterized by rising temperatures and altered precipitation patterns, enhances the risk of *A. mearnsii* invasion in Asia, making it crucial to identify high-risk areas for effective management. This study performed species distribution modeling using the maximum entropy (MaxEnt) algorithm to predict the potential introduction and spread of *A. mearnsii* under various climate scenarios based on shared socio-economic pathways (SSP2-4.5 and SSP5-8.5). Currently, only 4.35% of Asia is invaded, with a high invasion risk identified in six countries, including Bhutan, Lebanon, and Taiwan, where more than 75% of their areas are threatened. Under future climate scenarios, 21 countries face invasion risk, among which 14 countries, such as Georgia, Laos, Republic of Korea, and Turkey, are at moderate to very high risk, potentially encompassing up to 87.89% of their territories. Conversely, Northern Asian countries exhibit minimal changes in invasion risk and are considered relatively safe from invasion. These findings underscore that climate change will exacerbate invasion risks across Asia, emphasizing the urgent need for robust management strategies, including stringent quarantine measures and control efforts, to mitigate the threat of *A. mearnsii* expansion.

## 1. Introduction

Invasive plants are nonnative alien species, intentionally or inadvertently introduced, that establish self-sustaining populations, invade native natural ecosystems, and disturb agrobiodiversity [1,2]. Additionally, invasive plants homogenize plant composition, threaten rare and unique species, disrupt ecosystem stability, and cause substantial social and financial losses [3]. Anthropogenic climate change further exacerbates the problem because most invasive plant species respond positively to rising temperatures, increased nitrogen accumulation, elevated CO_2_ levels, and erratic precipitation regimes [4,5].

Global annual temperature has increased at an average rate of 0.06 °C per decade since 1850; since 1982, the rate of change in temperature has increased by 0.20 every 10 years, and the average global temperature maximum in 2023 was 13.9 °C [6]. Similarly, the rate of temperature increase in Asia is much higher than the global average temperature, and was 0.41 °C between 1982 and 2023 [6]. The state of the climate in Asia 2023 report emphasized the rapid increase in critical climate change indicators, including surface temperature, glacier retreat, and sea level rise, which will significantly increase the risk of invasive species and their negative impact on native biodiversity, agricultural productivity, and local economies [7].

Global trade, involving the import of agricultural and horticultural products and plants for pasture development, and the prevention of soil erosion and international tourism, further exacerbate the risk of the potential introduction and spread of invasive alien species worldwide, including in Asia [8,9,10,11]. Many parts of Asia, especially Southeast Asia and South Asia, are considered biodiversity hotspots that are already threatened by invasive alien plants such as *Mimosa pigra*, *Chromolaena odorata*, *Parthenium hysterophrous*, *Lantana camara*, and *Mikania micrantha* [12,13,14]. Our previous study showed that Asia and Europe will be new invasion hotspots by 2061–2080 if the current greenhouse gas emissions continue [15]. Interactions between biological invasion and climate change can magnify their effects, emphasizing the need for comprehensive studies on invasive species that may invade regions, in order to develop effective prevention measures.

*Acacia mearnsii*, a Leguminosae tree native to Southeast Australia, has been introduced and naturalized globally due its economic value. It was introduced to South Africa, North America, Europe, and Asia in the 1820s–1900s, primarily for tanbark, timber, soil conservation, and afforestation [16,17]. This species is listed as one of the world’s worst invasive species due to its rapid growth, prolific seed production, and ability to displace indigenous vegetation [16,18].

*A. mearnsii* can survive in wide temperature and precipitation ranges of 14.7 °C to 27.9 °C and 800 to 150 mm, respectively, and can grow in various soil types, including acidic to neutral, deep fertile, and moist soils [19]. Therefore, it can adapt to different environments, and it produces abundant seeds that can remain viable in soil for ~60 years, staying dormant until triggered by events such as wildfires, which lead to high germination rates and dense stands that outcompete native vegetation through shading [20,21,22]. Intentional introductions for agroforestry and tannins have facilitated its invasion across different countries of continental Asia [22]. This poses a significant threat to natural ecosystems as it forms dense, evergreen thickets, displacing native plant diversity [23].

Thus, to prevent invasion and conserve native biodiversity, it is essential to understand how *A. mearnsii* may colonize, establish, and spread under climate change. Species distribution models (SDMs) have been employed to predict alien and invasive plant species [24]. SDMs integrate species-occurrence data with environmental data layers to infer ecological requirements and potential habitat suitability for a species across a geographic area [25,26]. Among the various SDMs, the maximum entropy (MaxEnt) model is a popular machine learning algorithm widely used in ecology and conservation biology for predicting species distributions based on presence-only data and environmental variables due to its reliable results, short operation duration, and accurate prediction ability [27,28].

Predicting existing and potential future distributions of *A*. *mearnsii* and assessing the primary environmental variables impacting its distribution are important for the responsible management, utilization, and conservation of resources. In the present study, global species-occurrence records for *A. mearnsii* were used to estimate its invasion risk in different countries of Asia, and to predict habitat suitability based on bioclimatic variables under the current and potential future shared socio-economic pathway (SSP) climate change scenarios (SSP2-4.5 and SSP5-8.5). The primary objectives were (1) to identify the main environmental variables that affect the distribution of *A. mearnsii* in Asia; (2) to predict the current (1979–2013) and potential future (2041–2060 and 2081–2100) distributions of *A. mearnsii* under climate change scenarios SSP2-4.5 and SSP5-8.5 in Asia; and (3) to classify *A. mearnsii* habitat suitability in countries of Asia.

## 2. Results

### 2.1. Modeling Variables and Model Performance

Of nineteen bioclimatic variables, we selected six based on their weak correlation with each other (r < 0.75) through Spearman’s correlation analysis (Table 1). These variables included annual mean temperature (Bio1), mean diurnal temperature range (Bio2), isothermality (Bio3), annual precipitation (Bio12), precipitation in the wettest month (Bio13), and precipitation in the driest month (Bio 14). These selected variables are considered to be the most influential factors for accurately predicting the worldwide distribution of *A. mearnsii*. The average contribution of each variable to the model for current (1979–2013) and future (2041–2060 and 2081–2100) time periods shows that Bio3, Bio14, and Bio1 make the largest contributions at 51.72%, 19.74%, and 19.64%, respectively (Table 1). Similarly, Jackknife tests also indicate that Bio1, Bio3, and Bio12 were identified as key variables in predicting the potential distribution range of *A. mearnsii* (Figures S1–S5). Model performance was assessed using AUC values, and AUC values obtained from the rarefied species-occurrence points had higher scores (0.926) than those obtained from all occurrence points (0.832). These results suggest that predictions from rarefied species-occurrence points are more accurate and less prone to overestimation.

### 2.2. Distribution of A. mearnsii in Asia under Current Climate (1979–2013)

**Figure 1:**
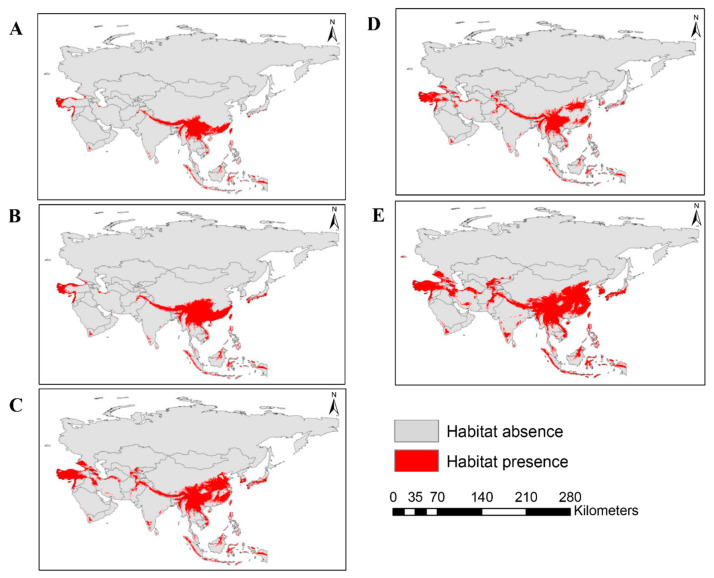
Distribution of *A. mearnsii* in Asia under the current and future (SSP2-4.5) climatic scenarios for (**A**) current (1979–2013), (**B**) SSP2-4.5 (2041–2060), (**C**) SSP2-4.5 (2061–2080), (**D**) SSP5-8.5 (2041–2060), and (**E**) SSP5-8.5 (2081–2100) time periods. In the legend, gray indicates the absence of habitat and red indicates the presence of habitat suitable for *A. mearnsii*.

**Figure 2:**
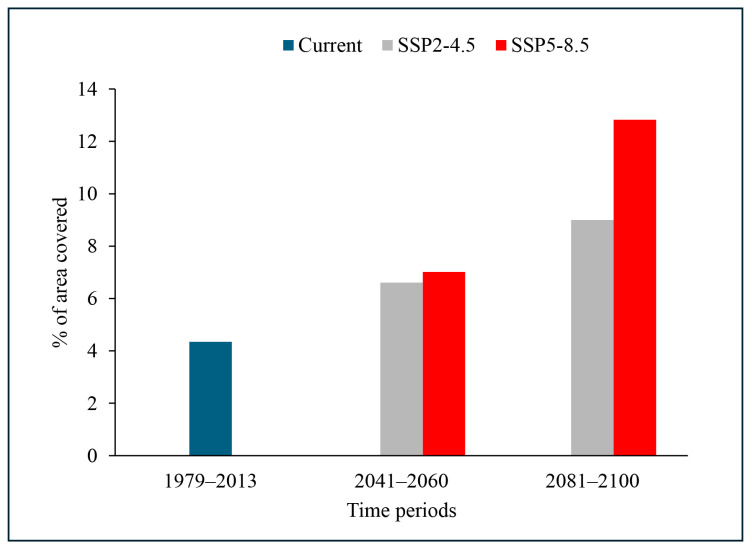
A graphical representation of the percentage area covered by *A. mearnsii* in Asia under the current and future climate change scenarios with the shared socio-economic pathways SSP2-4.5 and SSP5-8.5 for the 1973–2013, 2041–2060, and 2081–2100 time periods. Each cell has a 2.5 min resolution, which is approximately equal to 4.5 km at the equator.

The current distribution of *A. mearnsii* predicted by the MaxEnt model is presented in Figure 1A. The current distribution of *A. mearnsii* across Asia covers 149,325 cells, equivalent to 3,023,831 km^2^, which represents 4.35% of the total land surface (Figure 2). The distribution of *A. mearnsii* spans 32 countries of Asia, including China, Georgia, Israel, and Bhutan. Among them, Bhutan, Lebanon, and Nepal exhibit the highest area coverage of *A. mearnsii*, estimated to be 95.26%, 78.84%, and 71.34%, respectively (Table S1).

### 2.3. Potential Distribution of A. mearnsii under Future Climate Change Scenarios

We predicted the future spatial distribution of *A. mearnsii* for the periods 2041–2060 and 2081–2100 under two (SSP2-4.5 and SSP5-8.5) climate change scenarios. Our analysis showed that climate change will significantly expand its coverage, with a notable increase in suitable habitat across Asia (Figure 1B–E). On reaching the period 2081–2100, under the SSP2-4.5 scenario, its distribution is expected to span 309,117 cells, covering an area of approximately 6,259,619 square kilometers. This represents 9% of Asia’s total land area (Figure 2). In contrast, the more extreme SSP5-8.5 scenario shows a considerably larger spread. Under this scenario, *A. mearnsii*’s distribution is projected to cover 440,651 cells, equivalent to about 8,923,183 square kilometers, or 12.83% of Asia’s land mass (Figure 2, Table S1). Countries such as Azerbaijan, Georgia, Japan, Republic of Korea, Tajikistan, and Turkey, which currently have little or no suitable habitat, are expected to gain more suitable habitat than other countries, with a significant risk of expansion by the year 2100. Among these, Republic of Korea has the highest predicted rate of expansion, with its current low suitable area of 0.16% projected to increase to 52.53% under SSP2-4.5 by 2081–2100, and up to 87.90% under SSP5-8.5 for the same period (Table S1). These findings highlight the considerable potential range expansion of *A. mearnsii* across Asia driven by global climate change.

### 2.4. Assessment of Mean Habitat Suitability of A. mearnsii across Various Countries in Asia

**Figure 3:**
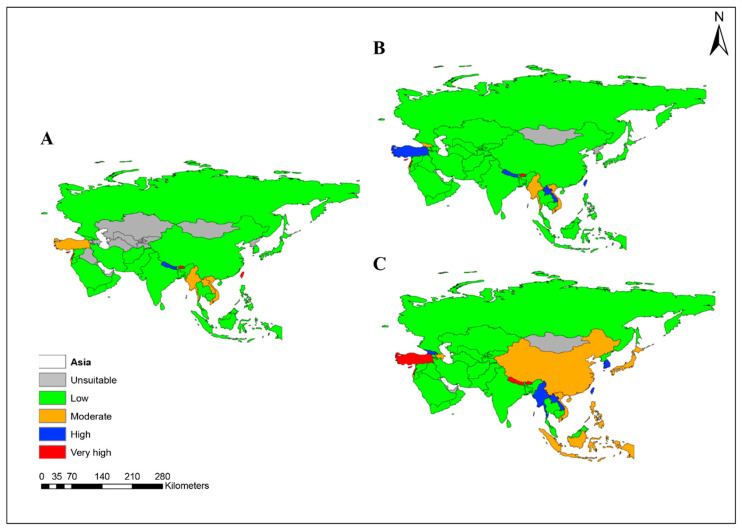
Mean habitat suitability of *A. mearnsii* in Asia under current and future climate change scenarios with the shared socio-economic pathways SSP 2-4.5 and SSP5-8.5. (**A**) Current time period (1979–2013), (**B**) average of SSP2-4.5 and SSP5-8.5 (2041–2060 time period), and (**C**) average of SSP2-4.5 and SSP5-8.5 (2081–2100 time period).

**Figure 4:**
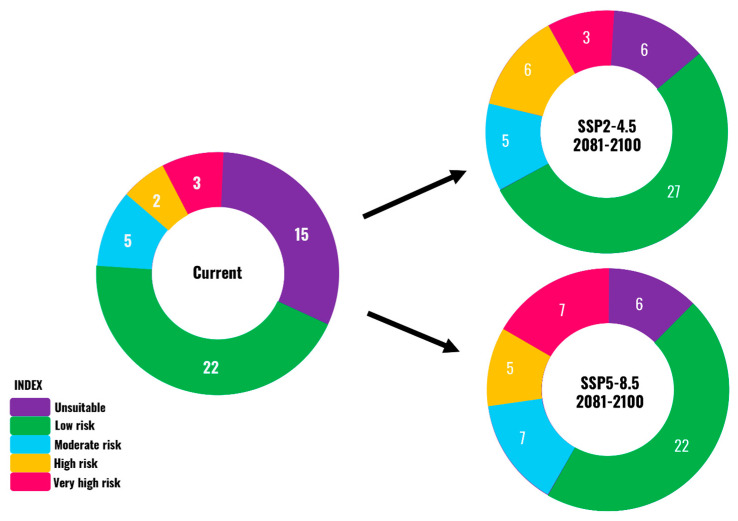
Pie charts showing the number of countries in Asia for which the risk of *A. mearnsii* invasion changes under the current and future climate change scenarios based on different risk categories.

The average invasion risk for *A. mearnsii* was assessed across all Asian countries, with each country categorized into five risk levels (unsuitable, low, moderate, high, and very high) under the current and future (SSP2-4.5 and SSP5-8.5) climate scenarios (Figure 3). Currently, 15 countries are unsuitable for *A. mearnsii* (Figure 4). However, climate change projections for the period 2081–2100 suggest a shift in its suitability. Under the SSP2-4.5 scenario, the number of unsuitable countries will decrease to 6, while the number of countries at high or very high risk will increase. Under SSP5-8.5, the number of unsuitable countries will remain at 6, but the risk levels will escalate significantly, with more countries classified as at high or very high risk (Figure 4).

Currently, 108,204 cells, equivalent to 2,191,131 km^2^, are under moderate risk of invasion, compared with 8115 (164,328.75 km^2^) at high risk, and 5009 (101,432.25 km^2^) at very high risk (Table 2). By 2081–2100, the high and very high-risk categories are estimated to increase by 824.19% and 1426.25%, respectively. Similarly, all countries of Asia were classified into five risk categories, and the changes in invasion risk categories under future climate change were predicted. The results showed that the rate of change in the invasion risk categories affects 21 countries (Table 3). Among them, 14 countries have a moderate to very high risk of invasion, with up to 87.89% of their area potentially invaded. Six countries, including Azerbaijan, China, and Tajikistan, are at moderate risk of invasion; four countries, including Georgia, Japan, and Vietnam, are at high risk of invasion; and four countries, including Laos, Nepal, and Republic of Korea, are at very high risk of invasion (Table 3).

## 3. Discussion

This study aimed to predict the risk of *A. mearnsii* invasion in different countries of Asia. Among the 19 bioclimatic variables, isothermality is the most important variable determining the distribution of *A. mearnsii* (Table 1). The distribution of *A. mearnsii* was estimated to be 4.35% of the total land surface of Asia, but the model under the SSP2-4.5 and SSP5-8.5 scenarios predicted increased invasion risk up to 9.00% and 12.83%, respectively, by 2081–2100 (Figure 2). Currently, 15 countries and ~12.14% of the land surface of Asia are unsuitable for invasion by *A. mearnsii*, but by 2081–2100, invasion risk is predicted to increase from low to high in 21 countries, and from low to very high in Laos, Nepal, Republic of Korea, and Turkey (Table 3 and Table S2).

Global climate change, driven primarily by human activities that release greenhouse gasses into the atmosphere, is one of the most pressing environmental challenges facing our planet [29,30]. Decades of scientific research and observations have provided overwhelming evidence of Earth’s rapidly changing climate. One of the most significant indicators of climate change is the rise in global surface temperature. According to the Intergovernmental Panel on Climate Change (IPCC) [29], Earth’s average surface temperature has risen by ~1.1 °C since the late 19th century, with the past decade (2011–2020) being the warmest on record globally [31]. This temperature rise is largely attributed to increased concentrations of greenhouse gasses, including carbon dioxide, methane, and nitrous oxide, resulting from human activities such as burning fossil fuels, deforestation, and agriculture [32]. If greenhouse gas emissions continue unabated, the IPCC (2021) projects that global temperature could rise by 2.6 °C to 4.8 °C by the end of this century, compared with pre-industrial levels. This would lead to even more severe and potentially irreversible impacts on Earth’s climate system, ecosystems, and human societies. In Asia, climate change-related risks are projected to increase progressively at 1.5 °C, 2 °C, and 3 °C of global warming in many parts of the continent [33].

Heat stress and water deficits are already affecting human health and food security [34]. Risks due to extreme rainfall and sea level rise are exacerbated in vulnerable regions of Asia. Particularly high average temperatures were recorded from Western Siberia to Central Asia, and from Eastern China to Japan. Japan and Kazakhstan have recently recorded their warmest years [7]. Global warming, precipitation and Asian monsoon alterations, permafrost thawing, extreme events such as dust storms, and the interplay between natural and human-related factors modify natural vegetation and increase the risk of the survival and spread of invasive weeds originating from tropical climates [7].

Climate change may create favorable conditions for invasive plants to expand their ranges into previously unsuitable areas, allowing them to outcompete native species adapted to historical climatic conditions [35,36]. To effectively address the threat posed by alien and invasive species, it is crucial to proactively predict their potential for introduction and dispersal using standardized modeling techniques. The MaxEnt model is commonly used to predict habitats of invasive plants due to its effectiveness in handling presence-only data [37]. It estimates habitat suitability by maximizing entropy subject to constraints, making it suitable for situations where only occurrence data are available [27,28]. Therefore, the MaxEnt model is used globally for predicting invasive species [37,38,39].

Asia is a large continent with diverse climates due to its varied topography, latitude, and geographical features. The southern and western parts of Asia, including India, Iran, and Pakistan, experience mild to hot climates up to 54 °C on summer days, while far northeastern areas such as Siberia in Russia are extremely cold with minimum temperatures down to −68 °C in winter [40,41,42]. Meanwhile, East Asian countries, including Japan, Korea, and China, experience a monsoonal climate characterized by hot and rainy summers. These climatic characteristics and increased human activities, such as transportation, create ideal conditions for the spread of invasive species [43,44]. Therefore, we studied the invasion risk of *A. mearnsii* in Asia under changing climate scenarios.

*A. mearnsii* grows fast and adapts to different climates. It was introduced to Asia primarily for commercial uses, including timber harvesting, tannin production, the pulp industry, and firewood production. In the last decade of the 18th century, *A. mearnsii* was introduced to the Indomalayan region of Asia and planted on a commercial scale in mountainous areas of Java, Southern Sulawesi, Sumatra, Bali, Peninsular Malaysia, Southern China, India, Vietnam, and the Philippines [45]. *A. mearnsii* has now reached several other countries of Asia. Our model showed that it is present in 33 countries under the current climate conditions, covering ~4.35% of the total land surface of Asia (Figure 2). The rate of invasion of *A. mearnsii* is very high due to the production of large numbers of seeds that can be dispersed over large distances via several mechanisms involving water, mammals, and birds [46]. It causes several environmental problems and is hard to control due to its ability to form root suckers. *A. mearnsii* competes aggressively for resources such as water, soil nutrients, and nitrogen, thereby impeding the growth of native plants [47,48]. Areas invaded by this plant can accumulate large amounts of leaf litter, making it challenging for native seedlings to thrive in such conditions [20,49]. Therefore, it is listed as one of the world’s 100 worst invaders [46].

Global climate change increases the risk of the invasion of nonnative species worldwide. The invasion risk of *A. mearnsii* is expected to increase in East Asia and parts of South and West Asia (Figure 1), potentially covering ~12.82% of the area by the end of this century under the SSP5-8.5 scenario. The rate of change in invasion risk was studied in each country of Asia, revealing that invasion risk will increase in at least 21 countries, particularly in East Asian countries such as Republic of Korea, Japan, Laos, Myanmar, and Vietnam, which are at high to very high risk of invasion (Figure 4, Table 4). By contrast, North Asian countries, including Russia and Mongolia, are relatively safe from invasion risk. These results indicate that East Asian countries with warm and sub-humid to warm humid climates are climatically suitable for *A. mearnsii*, resulting in a higher risk of invasion than northern regions with extreme cold and xeric environments.

*A. mearnsii* can survive at mean maximum temperatures of 21–27 °C, but cannot tolerate minimum temperatures below −3 to −7 °C because frost can damage its foliage and seeds, as reported in China [50]. *A. mearnsii* is considered an invasive species in many regions due to its aggressive spread and allelopathic properties, which can inhibit the growth of other plants [51]. Its foliage and seeds can be toxic to livestock and wild herbivores, causing digestive issues and potential fatalities [52]. Additionally, it has the ability to alter soil nutrients, particularly by increasing nitrogen levels and acidifying the soil [53]. Despite its detrimental effects, *A. mearnsii* has been studied for its medicinal properties and bioactive compounds, which have antimicrobial, antioxidant, and anti-inflammatory effects. Additionally, compounds such as tannins, flavonoids, and polyphenols have been identified in *A. mearnsii*, potentially contributing to its therapeutic potential [54,55]. Various methods have been employed to control the spread of *A. mearnsii*, including mechanical removal, burning, and the use of herbicides. However, these methods can be labor-intensive and may have unintended environmental consequences [56]. Biological control agents, such as seed-feeding insects and pathogens, have been explored as potential solutions [57]. A strict quarantine system at borders between countries is needed to effectively manage and regulate the spread of *A. mearnsii*, mitigate its potential adverse consequences, and facilitate the recovery of indigenous plant species.

## 4. Materials and Methods

### 4.1. Occurrence Data

**Figure 5:**
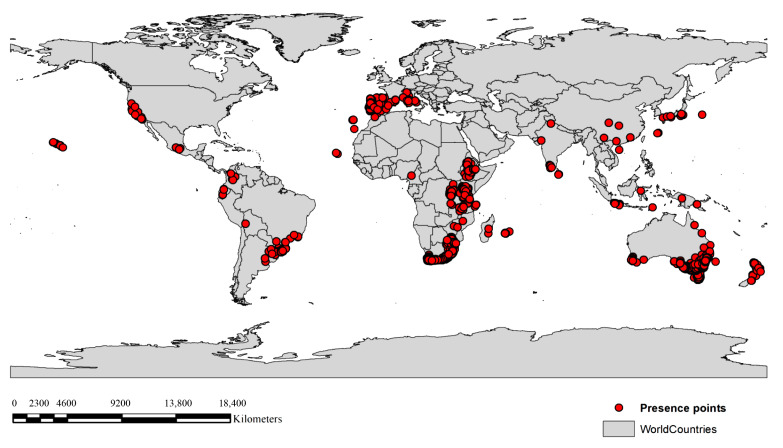
Global occurrence points for *Acacia mearnsii*. Red points represent worldwide occurrence points for *A. mearnsii*.

In this study, 25,953 occurrence records for *A. mearnsii* were collected from Global Biodiversity Information Facility (GBIF) open source data (https://www.gbif.org, accessed on 13 July 2023). To prevent duplicate coordinates and sampling biases, we utilized the spatially rarefy occurrence tool in the ArcGIS SDM toolbox v.2.4 [58] to select occurrence points, retaining only a single observation within each 2.5 min resolution grid cell, as described previously [38]. Ultimately, occurrence record points were reduced to 5284 (Figure 5), providing sufficient data points for use in MaxEnt modeling (Table S3).

### 4.2. Environmental Variables

Current climatic data (1979–2013) consisting of 19 bioclimatic variables at a 2.5 min resolution were downloaded from PaleoClim V1.2 (http://www.paleoclim.org, accessed on 23 July 2023) [59]. Future climatic data spanning two time periods (2041–2060 and 2081–2081) under the SSP2-4.5 and SSP5-8.5 scenarios were downloaded from WorldClim (http://worldclim.org, accessed on 7 August 2023) and used to build models for predicting *A. mearnsii* distribution at the global scale. Bioclimatic variables were generated by the global Beijing Climate Center Climate System Model (BCC-CSM2-MR) [60] under the Coupled Model Intercomparison Project (CMIP6) at a spatial resolution of 2.5 min (4.5 km at the equator). This bioclimatic model is widely used in Asia (e.g., China) [61] and was used to predict the geographical distribution of *A. mearnsii* in this study.

SSPs are sets of scenarios that explore potential future trajectories of global societal development, considering factors such as population growth, economic trends, technological progress, and environmental challenges [62]. Climate models predict that under the SSP2-4.5 and SSP5-8.5 scenarios, the global average temperature could rise by 1.8–4.1 °C and 3.8–8.6 °C, respectively, compared with pre-industrial levels [63]. The WorldClim portal is commonly used to understand these potential impacts on species distributions in response to changes in temperature and precipitation [64]. These variables, including annual mean temperature, precipitation pattern, and temperature seasonality, are crucial for modeling the ecological niches and potential range shifts of species in response to climate change [65].

To improve the accuracy of species distribution models, selecting bioclimatic variables that are not highly correlated with each other is important because multicollinearity can lead to biased parameter estimates and unreliable predictions [66]. A Pearson’s correlation analysis was conducted to identify bioclimatic variables with low correlation (r > 0.75, *p* = 0.05) and eliminate highly correlated ones, as described previously [67] (Table S4). Among the 19 bioclimatic variables examined (Table S5) six were selected, including temperature-related variables such as annual mean temperature (Bio1), mean diurnal temperature range (Bio2), isothermality (Bio03), and precipitation-related variables, including annual precipitation (Bio12), precipitation in the wettest month (Bio13), and precipitation in the driest month (Bio14). This approach has been widely employed in previous studies [68,69,70]. Pearson’s correlation analysis was conducted using the PROC CORR function of SAS 9.4 (SAS Institute Inc., Cary, NC, USA).

### 4.3. Model Development

**Figure 6:**
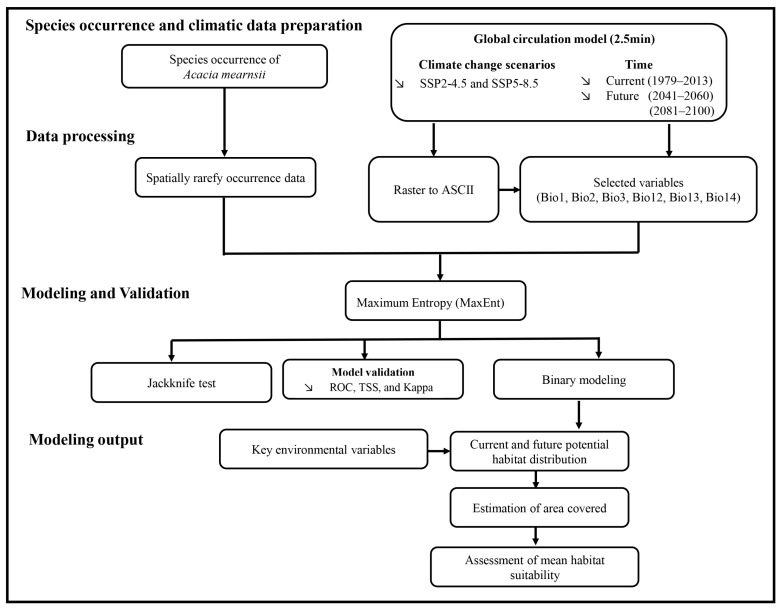
A flow chart showing the processing methodology and an overview of the entire workflow.

The global distribution of *A. mearnsii* under both the current and projected future climate change conditions was predicted using MaxEnt Version 3.4.1 [28]. MaxEnt, an open-source and widely applied machine learning technique, demonstrates robust predictive accuracy despite being trained on limited presence-only datasets [27,71]. MaxEnt is the most suitable tool for modeling invasive species because absence data for such species are typically scarce, and may be unreliable due to their expanding ranges and potential lack of saturation in available data [72]. Therefore, we identified global background points using ArcGIS 10.8 (ESRI, Redlands, CA, USA) as described in previous studies [69,73,74]. To assess the model’s predictive performance, species-occurrence data were randomly divided into a 3:1 ratio for model calibration and model validation as previously described [75]. The model was replicated 100 times, employing default parameters for all other MaxEnt options, to yield an average outcome [39]. The entire methodology and a processing overview of the workflow are illustrated in Figure 6.

### 4.4. Evaluating and Validating Model Results

Model performance was assessed using three distinct statistical measures. The first was area under the curve (AUC) values from receiver operator characteristic (ROC) curve analysis (Pearson 2010), where an AUC from 0 to 1 serves as a threshold-independent evaluation of model performance with the following categories: fail (0.5–0.6), poor (0.6–0.7), fair (0.7–0.8), good (0.8–0.9), and excellent (0.9–1) [76,77]. ROC curve analysis examines the effectiveness of the MaxEnt Model by determining its prediction accuracy based on AUC value, or the area enclosed by the curve. The jackknife test is a valuable technique used in MaxEnt modeling to assess the importance of individual environmental variables for SDMs. In the MaxEnt model, the jackknife test evaluates the contribution of each predictor variable by systematically excluding it from the model and measuring the resulting change in model performance [28]. Specifically, it assesses how much the model’s predictive accuracy (e.g., AUC-ROC) decreases when a particular variable is removed. Therefore, a jackknife test was conducted to ascertain the most important variables for predicting the potential distribution of targeted species [28].

### 4.5. Predicting Potential Habitat and Habitat Expansion of A. mearnsii in Asian Countries

Global binary distribution maps for *A. mearnsii* were determined using probability distribution maps produced from the MaxEnt model based on the maximum training sensitivity and the specificity Cloglog threshold. The binary distribution maps representing habitats in which *A. mearnsii* is present or absent were determined under the current and future (SSP2-4.5 and SSP5-8.5) climate change scenarios for the designated time periods 1970–2000, 2041–2060, and 2081–2100. The binary distribution maps for Asia for each time period were extracted via global binary distribution maps of *A. mearnsii* using the ‘extract by mask’ option of the spatial analyst tool in ArcGIS Desktop 10.8.

The mean habitat suitability for *A. mearnsii* in each country of Asia was estimated under the SSP2-4.5 and SSP-.8.5 climate change scenarios using the zonal statistics of the spatial analyst tool in ArcGIS, and the distributions of *A. mearnsii* during the current, 2041–2060, and 2081–2100 time periods were compared. The current and future invasion risk of *A. mearnsii* in Asian countries were evaluated based on mean habitat suitability, which was divided into five categories: unsuitable (0), low suitability (0.001–0.25), moderate suitability (0.26–0.50), high suitability (0.51–0.75), and very high suitability (0.76–1). The results revealed the threat of *A. mearnsii* invasion in different countries of Asia under the current and future climate change scenarios.

## 5. Conclusions

*A. mearnsii* is a highly invasive plant that threatens biodiversity and ecosystem functioning by altering soil nutrients, particularly by increasing nitrogen levels and acidifying soils. Global climate change increases the invasion risk of alien plants, including *A. mearnsii*. Species distribution modeling of *A. mearnsii* was performed to assess its invasion risk in different Asian countries under different climate change scenarios. The study revealed that currently, *A. mearnsii* is limited to ~4.35% of Asia’s total land surface, but this is projected to increase to ~12.82% by 2081–2100. Several countries, including Laos, Nepal, and Turkey, are at very high risk of invasion. Furthermore, an assessment of mean habitat suitability revealed that many East Asian countries, such as Republic of Korea and Japan, will become future invasion hotspots for *A. mearnsii*. Our study highlights the potential invasion risk posed by *A. mearnsii* in Asia. Our results underscore the urgent need for preventive measures, such as stringent quarantine protocols, to curb the introduction and spread of this detrimental weed. Our findings provide valuable insights for the development of long-term management strategies at both regional and local levels across Asian countries.

## Figures and Tables

**Table 1:** Selected bioclimatic variables and their average contributions in the model.

Variables	Variable Descriptions	Units	Model Contribution (%) ^a^
Bio1	Annual mean temperature	°C	19.64
Bio2	Mean diurnal temperature range	°C	0.55
Bio3	Isothermality (BIO2/BIO7; * 100)	%	51.72
Bio12	Mean annual precipitation	mm	8.28
Bio13	Precipitation in wettest month	mm	0.06
Bio14	Precipitation in driest month	mm	19.74

^a^ Average contribution of selected environmental variables in MaxEnt model for *A. mearnsii* under current and future (SSP2-4.5 and SSP5-8.5) climatic scenarios for 1979–2013, 2041–2060, and 2081–2100 time periods. Variables Bio1, Bio2, Bio3, Bio12, Bio13, and Bio14 represent annual mean temperature, mean diurnal temperature range, isothermality, annual precipitation, precipitation in wettest month, and precipitation in driest month, respectively.

**Table 2:** Changes in proportion of mean habitat suitability for *A. mearnsii* between current and future (SSP2-4.5 and SSP5-8.5) climate change scenarios by 2041–2060 and 2081–2100.

Risk to Countries	Current ^a^ (1979–2013)	Change in Risk Categories (%)			
		SSP2-4.5		SSP5-8.5	
		2041–2060	2081–2100	2041–2060	2081–2100
Unsuitable	417,059	−1.25	−73.31	−71.51	−73.31
Low	2,897,555	−0.55	9.29	9.96	7.18
Moderate	108,204	−5.41	−29.16	−47.13	−37.47
High	8115	238.28	284.93	769.43	824.19
Very high	5009	155.60	896.51	−36.57	1426.25

^a^ Numbers in columns represent cell number counted for different risk categories. SSP, shared socio-economic pathway.

**Table 3:** Predicted changes in risk of *A. mearnsii* invasion in different Asian countries under current and future (SSP2-4.5 and SSP5-8.5) climate change scenarios.

S.N	Countries	Unsuitable Habitat	Low Suitability	Moderate Suitability	High Suitability	Very High Suitability
1	Armenia	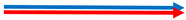				
2	Azerbaijan	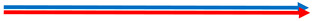				
3	China		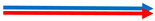			
4	Georgia		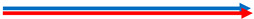			
5	Indonesia		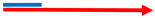			
6	Iraq	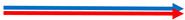				
7	Japan		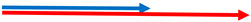			
8	Kazakhstan	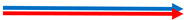				
9	Kyrgyzstan	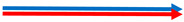				
10	Laos			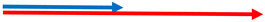		
11	Malaysia		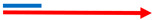			
12	Myanmar			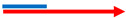		
13	Nepal				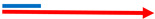	
14	North Korea	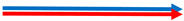				
15	Philippines		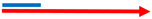			
16	Republic of Korea		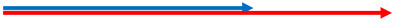			
17	Tajikistan	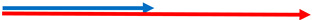				
18	Turkey			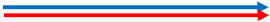		
19	Turkmenistan	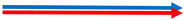				
20	Uzbekistan	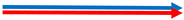				
21	Vietnam			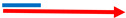		

Blue and red arrows in table indicate change in invasion risk between current (1979–2013) and 2081–2100 time periods under climate change scenarios SSP2-4.5 and SSP5-8.5, respectively, while straight lines represent constant risk level.

**Table 4:** Predicted changes in the risk of *A. mearnsii* invasion in different Asian regions under future (SSP2-4.5 and SSP5-8.5) climate change scenarios.

S.N	Regions	Unsuitable Habitat	Low Suitability	Moderate Suitability	High Suitability	Very High Suitability
1	East Asia		** 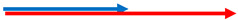 **			
2	Southeast Asia		** 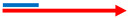 **			
3	West Asia		** 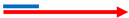 **			
4	Central Asia	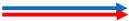				
5	North Asia			** 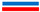 **		
6	South Asia			** 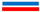 **		

Blue and red arrows in table indicate change in invasion risk between current (1979–2013) and 2081–2100 time periods under climate change scenarios SSP2-4.5 and SSP5-8.5, respectively, while straight lines represent constant risk level.

## Data Availability

No new data were created or analyzed in this study. Data sharing is not applicable to this article.

## Supplementary Materials

The following supporting information can be downloaded at: https://www.mdpi.com/article/10.3390/plants13202846/s1. Table S1: Percentage of area covered by *A. mearnsii* under current and future climate change scenarios (SSP2-4.5 and SSP5-8.5) for time periods 2041–2060 and 2081–2100; Table S2: Classification of countries according to average habitat suitability of current (1979–2013) and projected climate change scenarios SSP2-4.5 and SSP5-8.5 for time periods 2041–2060 and 2081–2100; Table S3: Occurrence points of *A. mearnsii* distributed worldwide; Table S4: Pearson correlation coefficients for determining most appropriate bioclimatic variables; Table S5: Environmental variables used in modeling of *A. mearnsii*; Figure S1: Jackknife test evaluation to assess test performance of *A. mearnsii* under current climate (1979–2013); Figure S2: Jackknife test evaluation to assess test performance of *A. mearnsii* under climate change scenario SSP2-4.5 for time period 2041–2060; Figure S3: Jackknife test evaluation to assess test performance of *A. mearnsii* under climate change scenario SSP2-4.5 for time period 2081–2100; Figure S4: Jackknife test evaluation to assess test performance of *A. mearnsii* under climate change scenario SSP5-8.5 for time period 2041–2060; Figure S5: Jackknife test evaluation to assess test performance of *A. mearnsii* under climate change scenario SSP5-8.5 for time period 2081–2100.
