# Supplementary material for: Predicting the Invasion Risk of the Highly Invasive Acacia mearnsii in Asia under Global Climate Change
Source: Plants (Basel). 2024 Oct 11;13(20):2846. doi: 10.3390/plants13202846 (PMC11510992; doi:10.3390/plants13202846)
Supplement: Supplementary file 1 [file plants-13-02846-s001.zip › Supplementary Figures.pdf]

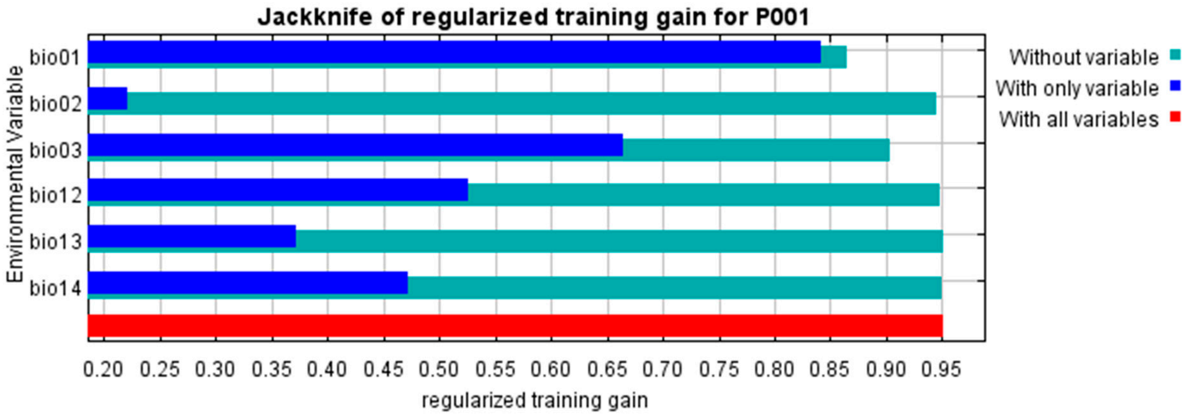

Figure S1. Jackknife test evaluation to assess test performance of *A. mearnsii* under the current climate (1979–2013).

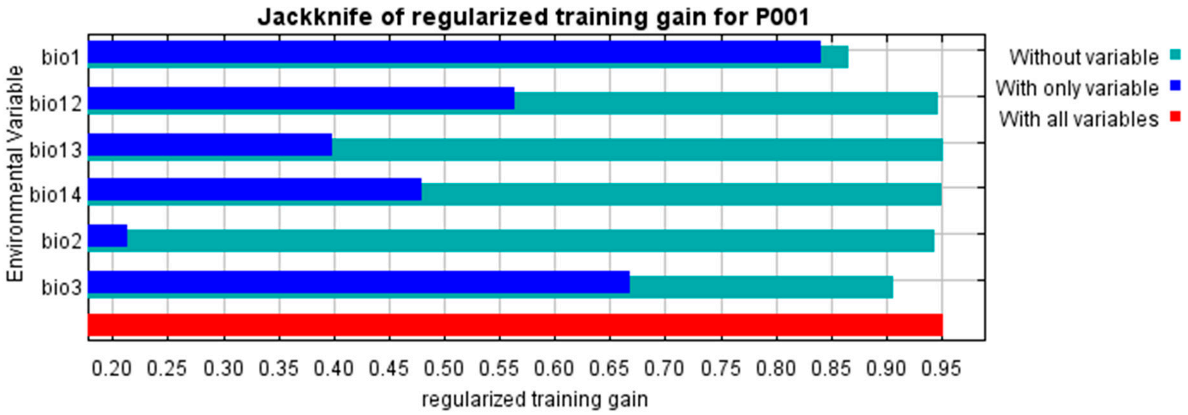

Figure S2. Jackknife test evaluation to assess test performance of *A. mearnsii* under the climate change scenario SSP2-4.5 for the time period 2041–2060.

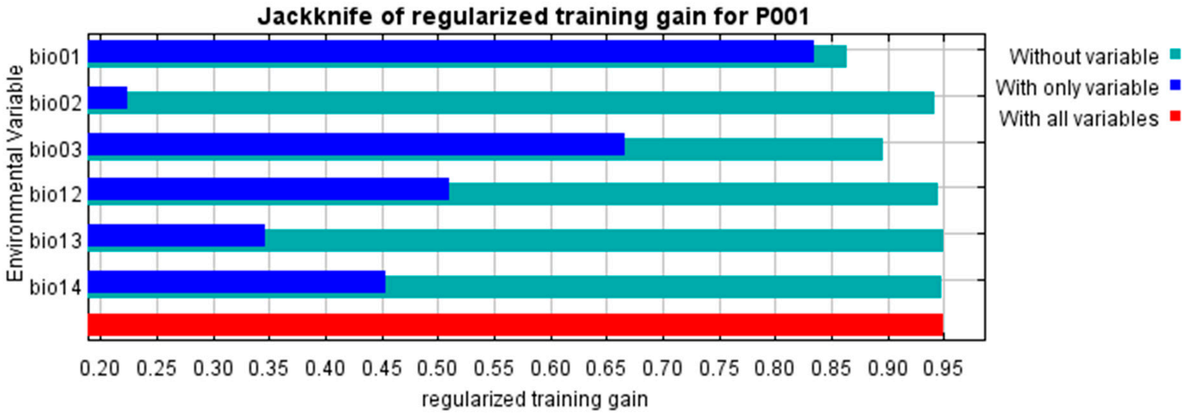

Figure S3. Jackknife test evaluation to assess test performance of *A. mearnsii* under under the climate change scenario SSP2-4.5 for the time period 2081–2100.

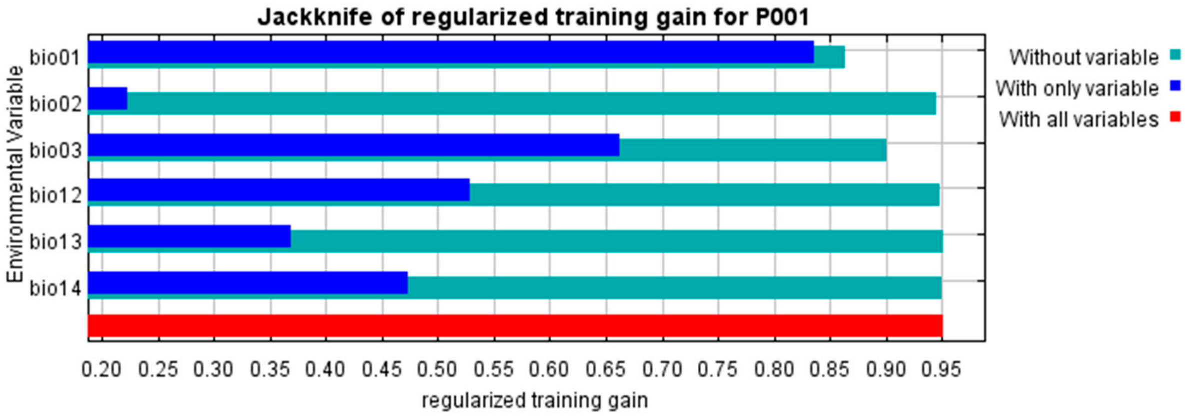

Figure S4. Jackknife test evaluation to assess test performance of *A. mearnsii* under the climate change scenario SSP5-8.5 for the time period 2041–2060.

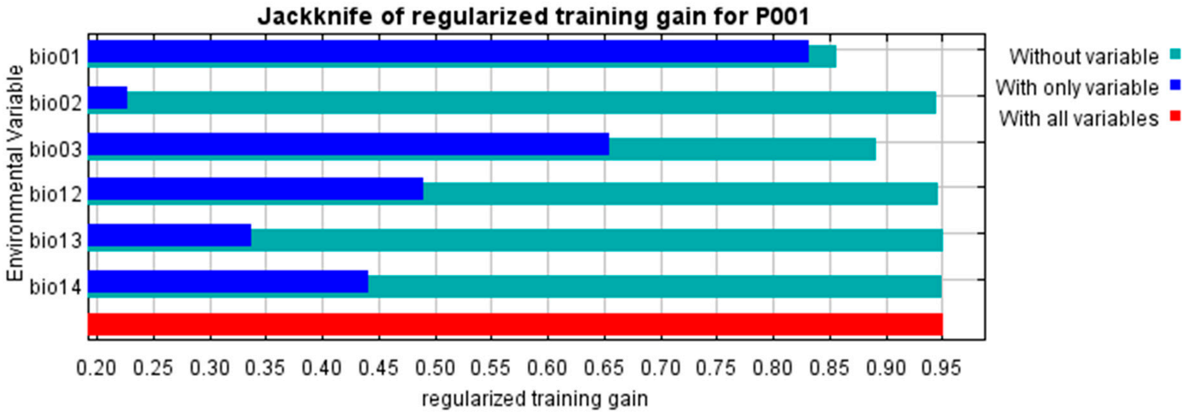

Figure S5. Jackknife test evaluation to assess test performance of *A. mearnsii* under the climate change scenario SSP5-8.5 for the time period 2081–2100.
